# CircSOD2 induced epigenetic alteration drives hepatocellular carcinoma progression through activating JAK2/STAT3 signaling pathway

**DOI:** 10.1186/s13046-020-01769-7

**Published:** 2020-11-25

**Authors:** Zhongwei Zhao, Jingjing Song, Bufu Tang, Shiji Fang, Dengke Zhang, Liyun Zheng, Fazong Wu, Yang Gao, Chunmiao Chen, Xianghua Hu, Qiaoyou Weng, Yang Yang, Jianfei Tu, Jiansong Ji

**Affiliations:** Key Laboratory of Imaging Diagnosis and Minimally Invasive Intervention Research, The Fifth Affiliated Hospital of Wenzhou Medical University /Affiliated Lishui Hospital of Zhejiang University/ Clinical College of The Affiliated Central Hospital of Lishui University, Lishui, 323000 China

**Keywords:** Hepatocellular carcinoma, Circular RNA, JAK2/STAT3, DNMT3a

## Abstract

**Background:**

Emerging evidence suggests that circular RNAs play critical roles in disease development especially in cancers. Previous genome-wide RNA-seq studies found that a circular RNA derived from SOD2 gene was highly upregulated in hepatocellular carcinoma (HCC), however, the role of circSOD2 in HCC remains largely unknown.

**Methods:**

The expression profiling of circSOD2 and microRNA in HCC patients were assessed by Real-Time Quantitative Reverse Transcription PCR (qRT-PCR). SiRNA or CRISPR-CAS9 were used to silence gene expression. The biological function of circSOD2 in HCC was investigated using in vitro and in vivo studies including, trans-well cell migration, cell apoptosis, cell cycle, CCK8, siRNA interference, western blots, and xenograft mouse model. The underlying molecular mechanism was determined by Chromatin Immunoprecipitation quantitative real time PCR (ChIP-qPCR), bioinformatic analysis, biotin-pull down, RNA immunoprecipitation, 5-mc DNA pulldown and luciferase assays.

**Results:**

In accordance with previous sequencing results, here, we demonstrated that circSOD2 was highly expressed in HCC tumor tissues compared with normal liver tissues. Mechanically, we showed that histone writer EP300 and WDR5 bind to circSOD2 promoter and trigger its promoter H3K27ac and H3K4me3 modification, respectively, which further activates circSOD2 expression. SiRNA mediated circSOD2 suppression impaired liver cancer cell growth, cell migration, prohibited cell cycle progression and in vivo tumor growth. By acting as a sponge, circSOD2 inhibits miR-502-5p expression and rescues miR-502-5p target gene DNMT3a expression. As a DNA methyltransferase, upregulated DNMA3a suppresses SOCS3 expression by increasing SOCS3 promoter DNA methylation. This event further accelerates SOCS3 downstream JAK2/STAT3 signaling pathway activation. In addition, we also found that activated STAT3 regulates circSOD2 expression in a feedback way.

**Conclusion:**

The novel signaling axis circSOD2/miR-502-5p/DNMT3a/JAK2/STAT3/circSOD2 provides a better understanding of HCC tumorigenesis. The molecular mechanism underlying this signaling axis offers new prevention and treatment of HCC.

**Supplementary Information:**

The online version contains supplementary material available at 10.1186/s13046-020-01769-7.

## Background

Hepatocellular carcinoma (HCC) accounts for > 80% of liver cancer cases and is the fourth leading cause of cancer-related death worldwide [1, 2]. The incidence of HCC varies between different regions with over 85% are estimated to occur in low or middle-resource countries, particularly in sub-Saharan Africa and Eastern Asia [3, 4]. Despite great advances have been made in the early diagnosis and treatment, the prognosis of HCC patients is still low due to late diagnosis, recurrence and late stage metastasis [5]. Dysregulation of signaling pathways including IL-6/STAT3, Wnt/β-catenin, and PI3K/AKT etc. [6–8] have been reported in HCC. However, the underlying molecular mechanism remains largely unknown. Identifying new molecular target or mechanism that drive HCC development will help us understand its pathogenesis and provide new therapeutic methods.

Recently, great attention has been paid to study the roles of non-coding RNAs in diseases, especially in cancer research [9–13]. Non-coding RNAs are groups of RNAs with little or no protein coding potency, including micro RNA (miRNA), long non-coding RNA (lncRNA), and recently emerged circular RNA (circRNA), etc. Circular RNA is defined by a continuous closed loop structure generated from its precursor mRNA through back splicing [14]. Although most circular RNAs do not encode protein, they can regulate gene expression in many ways. For example, by acting as a sponge, they can regulate miRNA expression [15, 16], through inhibiting RNAPII extension, they can regulate gene transcription [17]. In addition, circular RNAs were also reported to participate in a variety of other activities including gene alternative splicing process, histone modification, RNA maturation and protein synthesis, etc. [18–21]. Emerging evidence suggest that abnormal expression of circular RNAs have been implicated in the progression of diverse cancers including HCC [22–25].

Previous genome-wide RNA-seq studies on HCC tumor tissues and its adjacent normal liver tissues revealed that a circular RNA derived from SOD2 gene was highly expressed in tumor tissues compared to its adjacent normal liver tissues [26]. However, the role of circSOD2 in HCC remains unknown. Here, we found that highly expressed circSOD2 promotes liver cancer cell proliferation and is linked to cancer progression in vivo. Mechanically, we demonstrated that, upregulated circSOD2 suppressed miR-502-5p expression, which further promotes DNMT3a expression and activates JAK2/STAT3 signaling pathway. In addition, we also showed that STAT3 regulates circSOD2 expression in a feedback way. This study provided a new mechanism through which circSOD2 promotes HCC pathogenesis, and implicated new diagnostic and therapeutic targets.

## Material and methods

### Cell lines and patient sample

Cell lines used in this study including normal liver cell line HL-7702, liver cancer cell line HEPG2, HUH7, SK-HEP1 and HEP3B were purchased from Chinese Academy of Sciences, Shanghai, China. HEPG2 and SK-HEP1 were grown in MEM supplied with 10% Fetal Bovine Serum (FBS), HL-7702 and HUH7 were cultured in DMEM supplied with 10% FBS, HEP3B was grown in RPMI-1640 supplied with 15% FBS. All cells were maintained at 37 °C in a humidified incubator with 5% CO2. Primary tumor tissues and their paired adjacent normal tissues were obtained from patients who underwent surgery at Fifth Affiliated Hospital of Wenzhou Medical University. Samples were collected, snap freeze with liquid nitrogen and used for further studies. All patients gave written informed consent.

### Real-time quantitative reverse transcription PCR (qRT-PCR)

Cells or tissues were collected, total RNA was extract with Trizol reagent (Thermo Fisher, cat: 15596026). cDNA was generated with iScript™ Reverse Transcription Reagents (Bio-rad, cat: 1708841) following manufactures’ instructions. cDNA was detected with SYBG Super Real PreMix Plus (Tiangen, Cat: FP205). Relative RNA expression was calculated with 2-∆∆ct. Primers used in this study were listed in Table 1.

**Table 1:** Primers used in this study

Name	5′-3‘
qRT-circSOD2-F	AAACCACGATCGTTATGCTG
qRT-circSOD2-R	CGTTAGGGCTGAGGTTTGTC
CTCFsg-F	caccgCGATCCAAATTTGAACGCCG
CTCFsg-R	aaacCGGCGTTCAAATTTGGATCGc
STAT3sg-F	caccgAGATTGCCCGGATTGTGGCC
STAT3sg-R	aaacGGCCACAATCCGGGCAATCTc
EP300sg-F	caccgCTTGGCAAGACTTGCCTGAC
EP300sg-R	aaacGTCAGGCAAGTCTTGCCAAGc
WDR5sg-F	caccgCAGTGCCTGAAGACGCTCAT
WDR5sg-R	aaacATGAGCGTCTTCAGGCACTGc
DNMT3asg-F	caccgCCGCTCCGCAGCAGAGCTGC
DNMT3asg-R	aaacGCAGCTCTGCTGCGGAGCGGc
qRT-GAPDH-F	GGAGCGAGATCCCTCCAAAAT
qRT-GAPDH-R	GGCTGTTGTCATACTTCTCATGG
K27ac/K4me3-ChIP-qPCR-circSOD2p-F	GCCTATGAGCTGAGGGTAGA
K27ac/K4me3-ChIP-qPCR-circSOD2p-R	TGCCTCCTGTCCTGGAATA
pGL3-SOCS3p-F	ccGGTACCGGAGGCCGCGCTCGCGGG
pGL3-SOCS3p-R	aacAGATCTCGCGCAGCACCAAACTGCC
K27ac-SOCS3p-F	GCCTATGAGCTGAGGGTAGA
Me-SOCS3p-R	TGCCTCCTGTCCTGGAATA
EP300-ChIP-qPCR-circSOD2p-F	TGTGAACCAACTGTTCAGGATAA
EP300-ChIP-qPCR-circSOD2p-R	ACTGTTGAGAGAGCACTTGATAC
WDR5-ChIP-qPCR-circSOD2p-F	TTCCCACCTTCCTCACTACT
WDR5-ChIP-qPCR-circSOD2p-R	GGAAAGAATCCTCTGTTGTCCT
Me-qPCR-SOCS3p-F	CCCAACTTCTCATTCACACTTTC
Me-qPCR-SOCS3p-R	CAGGTCGGCCTCCTAGA
qRT-U6-F	GCTTCGGCAGCACATATACTAAAAT
qRT-U6-R	CGCTTCACGAATTTGCGTGTCAT.

### Luciferase assay

SOCS3 promoter was amplified with primers SOCS3p-F: ccGGTACCGGAGGCCGCGCTCGCGGG and SOCS3p-R: aacAGATCT CGCGCAGCACCAAACTGCC. Amplified fragment was inserted into PGL3-Basic vector between KpnI and BglII cutting sites. pcDNA3-myc-dnmt3a was purchased from BIofeng, china. 2μg PGL3-SOCS3p, 0.1μg Renilla vector, and different doses of PCDNA3-myc-dnmt3a were transfected into HEPG2 and HUH7 cells by Lipofectamine 2000. Cells were collected 48 h later. Luciferase activity was determined with Dual-Luciferase reporter assay kit (Promega, cat: E1910).

### Cell migration assay

Cell migration was performed in trans-well chambers (24-well plate, 8-μm pore size). In brief, cells were collected by trypsin digestion and washed with PBS twice. Load 100ul containing 1 × 105 cells onto the upper chamber and incubate the trans-well plate at 37 °C and 5% CO2 for 24 h. The lower compartment was added with 2 mL DMEM containing 1% FBS. 24 h later the upper chamber was removed, cells migrated to the lower chamber was fixed with 5% glutaraldehyde for 10 min at room temperature and stained with 0.1% Crystal Violet. Cell numbers in five widefield were counted following crystal violet staining.

### Cell proliferation assay

After transfection for 24 h, cells were collected, and approximately 1000 cells were seeded into 96 well plates. Cells growth was detected by CCK8 assay kit in accordance with manufacturer’s instructions. Relative vial cell number was evaluated by spectrofluorometer at wavelength OD450.

### Cell cycle

Cell cycle was measured with Propidium iodide (PI) staining. Cells were harvested, washed with PBS and fixed with 90% ice cold ethanol at − 20 °C overnight. The next day, cells were allowed to equilibrate to RT by incubating at RT for 5 min. Cells were then washed with PBS at RT twice and stained with 1 ml PI staining solution (50 μg/ml, 1 mg/ml of RNase A, 0.1% Triton X-100 in PBS) by incubating at RT for 30 mins in the dark. Flow cytometry was applied to detect cell cycle.

### Cell apoptosis

Cells were collected, washed with PBS and stained with 7-ADD and FITC labeled Annexin-V at ice for 30mins. After staining cell apoptosis was detected by FACS.

### CRISPR-CAS9 mediated gene knockout

EP300, WDR5, STAT3, DNMT3a, SOCS3 were deleted by CRISPR-CAS9. In brief, sgRNAs were designed with online tools (https://portals.broadinstitute.org/gpp/public/analysis-tools/sgrna-design). Annealed sgRNAs were cloned into pLentiGuide-Puro and co-transfected with VSVG, and PsPAX2 into 293 T cells with Mirus transfection reagent. Fresh medium was replenished~ 16 h later. Supernatant containing released lentivirus were collected and filtered through 0.45uM filter after 48 h. Collected lentivirus were used to infect target cells stably expressing CAS9. 2 days later, fresh medium containing 3μg/ml Puromycin was added into cells to deplete uninfected cells for another 3 days. The efficiency of gene knockout was evaluated by western blots.

### Chromatin Immunoprecipitation quantitative PCR (ChIP-qPCR)

Tissues were disrupted with dounce homogenizer to obtain single cell suspension. Cells were crosslinked with 1% formaldehyde and lysed with cell lysis buffer on ice for 20 min. DNA was then sonicated into 200 ~ 500 bp with bioruptor. Protein A/G beads were used for preclear followed by antibody pull down with shaking at 4 °C overnight. IgG was used as control. The next day, DNA-protein-antibody complexes were pulled down by Protein A/G beads. Beads were washed, DNA-Protein complexes were reverse crosslinked with Proteinase K, DNA was then purified with DNA purification kit. Quantification PCR was used to determine the enrichment of DNA.

### DNA methylation pull down (5-mc DNA pull down)

The methylation status on SOCS3 promoter was determined with Methylated-DNA IP Kit (Zymo research, Cat: D5101) according to manufacturer’s instructions. The enrichment of desired DNA region was evaluated by qPCR.

### RNA immunoprecipitation (RIP)

Cells were harvested and crosslinked with formaldehyde. Cells were then lysed, and chromatin was sonicated. Ago2 antibody or IgG was used to pull down Ago2-RNA complexes with shaking at 4 °C overnight. The next day, ProteinA/G beads was added to precipitate antibody-Ago2-RNA complexes. Beads were then washed to wash off unbound materials. RNA was purified with RNA purification kit. The enrichment of RNA was determined by qRT-PCR.

### In vivo xenograft mouse model

The animal experiment was strictly conducted based on the protocol of the institutional ethics board of the Fifth Affiliated Hospital of Wenzhou Medical University. Every effort was made to minimize the pain of mice. 4-week old BALB/C nude mice were purchased from shanghai animal center. siRNA targeting circSOD2 or scramble control siRNA was transfected into HEPG2 cells. Cells were collected 24 h later. 1 × 10^7^ cells resuspended in 200ul PBS were then subcutaneously injected into the right flanks of BALB/c nude mice. Tumor growth was monitored and measured. The experiment was stopped 5 weeks later.

### Western blots

Cells were harvested and lysed with Radioimmunoprecipitation assay buffer (RIPA buffer). Supernatants containing protein was obtained by centrifuging cell lysis at Maxi speed for 20 min. Protein was then boiled with 3XSDS loading buffer, separated on 9% SDS-PAGE gel and transferred to nitrocellulose membrane (NC). Primary and secondary antibody were used to detect protein of interest. Signals were visualized with ECL reagent.

### Statistic analysis

Data were analyzed with SPSS software. Two-side T test was used to determine the differences between untreated and treated groups. Cell growth and tumor formation were analyzed with Two way-ANOVA *P < 0.05, **P < 0.01, ***P < 0.001. *P* < 0.05 was considered statistically significant.

## Results

### CircSOD2 is highly expressed in HCC tumor tissues and liver cancer cell lines

**Fig. 1:**
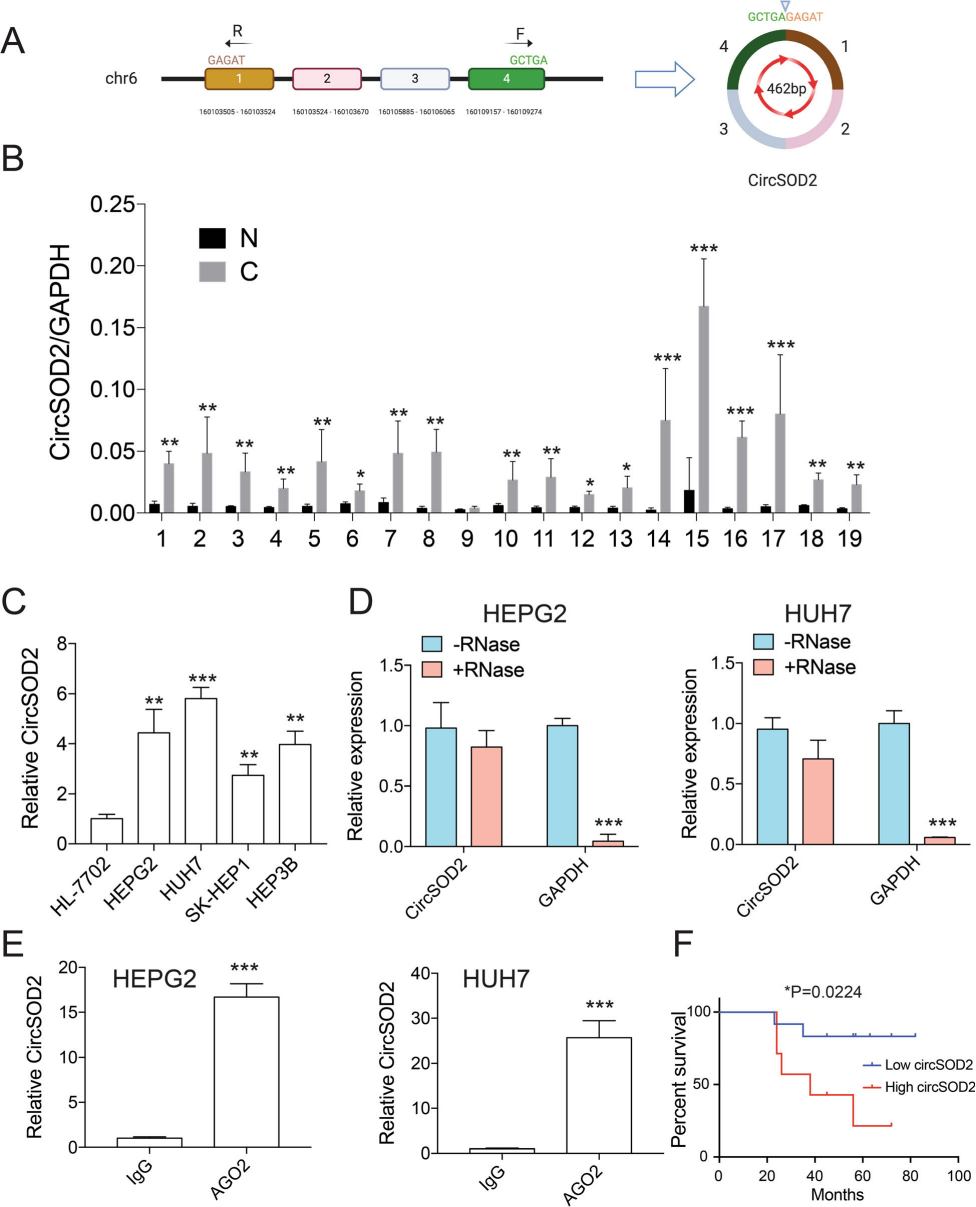
CircSOD2 is highly expressed in HCC tumor tissues and liver cancer cell lines. **a** Demonstration of circSOD2 formation, F and R are divergent primers used for circSOD2 detection. **b** RT-qPCR results of circSOD2 expression in 19 HCC patient tumor tissues and adjacent normal liver tissues. N normal, C cancer. **c** circSOD2 expression in normal liver cell HL-7702 and liver cancer cell line HEPG2, HUH7, SK-HEP1, and HEP3B. **d** circSOD2 expression after treatment with 1 mg/ml RNase A, GAPDH was used as control. **e** circSOD2 expression after pulling down with AGO2 or IgG. **f** The overall survival of HCC patients with low or high expression of circSOD2 in HCC tissues were assessed by Kaplan-Meier survival analysis. *P < 0.05, **P < 0.01, ***P < 0.001

Genome-wide RNA-seq studies on HCC tumor tissues and their adjacent nontumorous liver tissues revealed that hsa_circ_0004662, derived from SOD2 gene was significantly upregulated in HCC tumor tissues [26]. However, its role in HCC is still unknown. To validate RNA-seq results, qRT-PCR with divergent primers complementary to circSOD2 exon 1 and 4 (Fig. 1a) was used to detect circSOD2 expression from patients and cell lines. In line with RNA-seq data, circSOD2 expression was significantly upregulated in 18/19 HCC patient’s tumor tissues and liver cancer cell lines compared with normal liver tissues and normal liver cells (Fig. 1b-c). High circSOD2 expression was also associated with poor survival (Fig. 1f) and linked to higher grade tumors (Supplemental Table 1). To further characterize circSOD2, RNA was extracted from HEPG2 and HUH7 cells and treated with or without RNase A before reverse transcription. The effect of RNase A on circSOD2 expression was then examined. Similar to other circRNA, circSOD2 was highly resistant to RNase A digestion. However, the RNA level of GAPDH was greatly impaired after RNase A treatment (Fig. 1d). Data from circular RNA interactome showed that 13 circSOD2 binding sites exist in an RNA binding protein-Ago2 (data not shown), indicating circSOD2 may interact with Ago2 within the cell. To confirm this, RIP assay with Ago2 antibody or IgG control was performed. Indeed, Ago2 significantly precipitated circSOD2 from HEPG2 and HUH7 cells compared to IgG control (Fig. 1e).

### CircSOD2 promoter is intensively modified by H3K27ac and H3K4me3

**Fig. 2:**
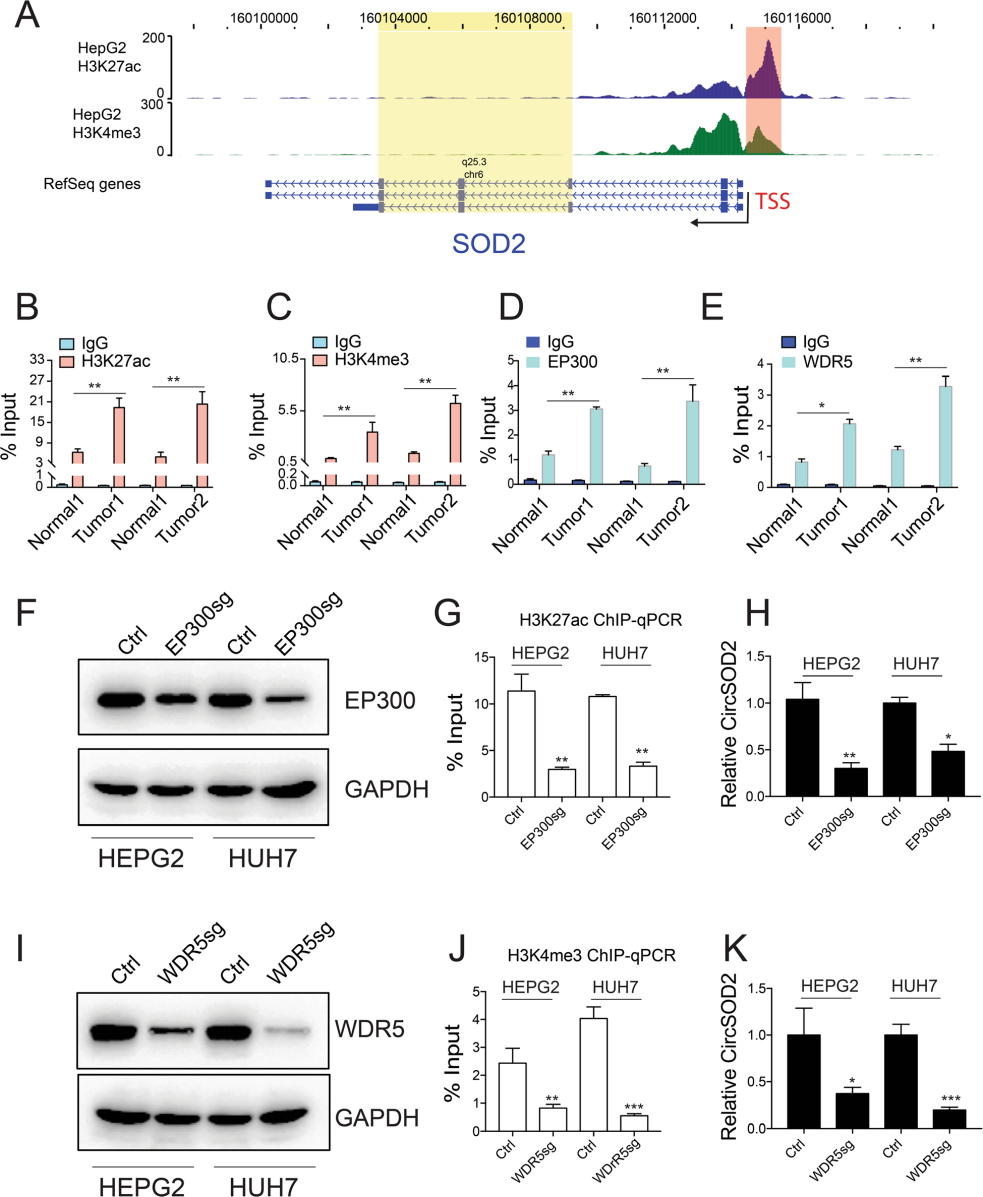
CircSOD2 promoter is intensively modified by H3K27ac and H3K4me3. **a** H3K27ac and H3K4me3 ChIP-seq results from HEPG2. circSOD2 and SOD2 promoter region were labeled with red, chromosome region spanning circSOD2 transcripts was labeled with yellow. H3K27ac (**b**) and H3K4me3 (**c**) ChIP-qPCR signal on circSOD2 promoter and control regions from HCC tumor tissues and adjacent normal liver tissues, IgG was used as control. Enrichment of EP300 (**d**) and WDR5 (**e**) on circSOD2 promoter and control regions from HCC tumor tissues and adjacent normal liver tissues. f Western blot results of EP300 after CRISPR-CAS9 mediated gene depletion, GAPDH was used as control. **g** H3K27ac signal on circSOD2 promoter after deleting EP300. **h** circSOD2 expression after deleting EP300. **i** Western blot results of WDR5 after CRISPR-CAS9 mediated gene depletion, GAPDH was used as control. **j** H3K4me3 signal on circSOD2 promoter after deleting WDR5. **k** circSOD2 expression after deleting WDR5. *P < 0.05, **P < 0.01, ***P < 0.001

H3K27ac and H3K4me3 modification indicate active gene transcription [27, 28]. To understand if these modifications contribute to circSOD2 upregulation in HCC. H3K27ac and H3K4me3 ChIP-seq data from HEPG2 cells were then examined. As shown in WashU browser, SOD2 promoter was extensively occupied by H3K27ac and H3K4me3 (Fig. 2a), correlating with high circSOD2 expression. ChIP-qPCR with H3K27ac and H3K4me3 antibodies confirmed higher H3K27ac and H3K4me3 modification on circSOD2 promoter from HCC tumor tissues and liver cancer cell lines compared to normal liver tissues (Fig. 2b-c) and normal liver cells (Supplementary figure 1A-B). EP300 and WDR5 mediate H3K27ac and H3K4me3 modification respectively. We next ask if EP300 and WDR5 bind to circSOD2 promoter. ChIP-qPCR was then performed. Similar to H3K27ac and H3K4me3 signal, EP300 and WDR5 were also significantly enriched in circSOD2 promoter from HCC tumor tissues compared to normal liver tissues (Fig. 2d-e). In addition, partial depletion of EP300 or WDR5 (Fig. 2f,i) greatly impaired H3K27ac or H3K4me3 signal on circSOD2 promoter (Fig. 2g,j), circSOD2 expression was also downregulated (Fig. 2h,k). Thus, these results indicate that the enrichment of EP300 and WDR5 on circSOD2 promoter increased its H3K27ac and H3K4me3 modification and circSOD2 expression in HCC.

### CircSOD2 promotes in vitro liver cancer cell proliferation and tumorigenesis in vivo

**Fig. 3:**
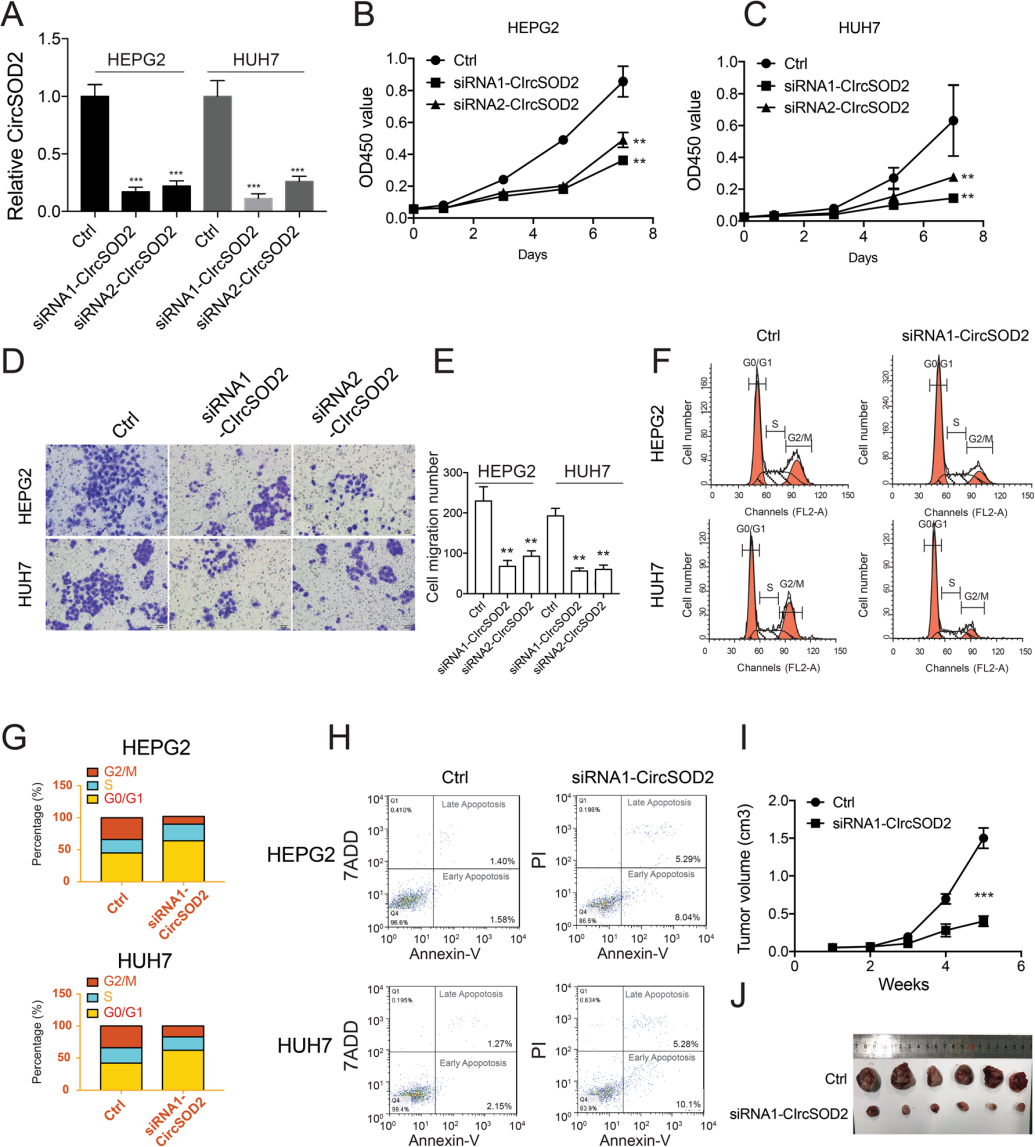
CircSOD2 promotes in vitro liver cancer cell proliferation and tumorigenesis in vivo. **a** CircSOD2 expression after silencing with siRNA. HEPG2 (**b**) HUH7 (**c**) Cell growth and cell migration (**d**) following knockdown of circSOD2. **e** Statistic results of cell migration. **f** Cell cycle following knockdown of circSOD2. **g** statistic results of F. **h** Cell apoptosis following knockdown of circSOD2. **i** CircSOD2 depleted from HEPG2 or HEPG2 control cells was injected into nude mice, tumor volume was monitored. **j** Mice was sacrificed 5 weeks later; tumor picture was taken. *P < 0.05, **P < 0.01, ***P < 0.001

To further characterize the role of circSOD2 in HCC, siRNA targeting the back spliced site of circSOD2 was used to silence circSOD2 expression in liver cancer cells. SiRNA efficiently silenced circSOD2 expression ~ 6 fold lower than its original level (Fig. 3a). CircSOD2 downregulation impaired HEPG2 and HUH7 cell growth, and cell migration (Fig. 3b-e). Moreover, cell cycle was also arrested in G0/G1 phase and cell apoptosis was increased following circSOD2 depletion (Fig. 3f-h). The role of circSOD2 in in vivo tumorigenesis was also examined. In accordance with impaired in vitro cell proliferation, silencing circSOD2 also decreased HCC tumor formation in nude mice compared with scramble control (Fig. 3i-j). Taken together, these results suggest that high circSOD2 expression may associate with HCC development.

### CircSOD2 suppresses miR-502-5p expression by acting as a sponge

**Fig. 4:**
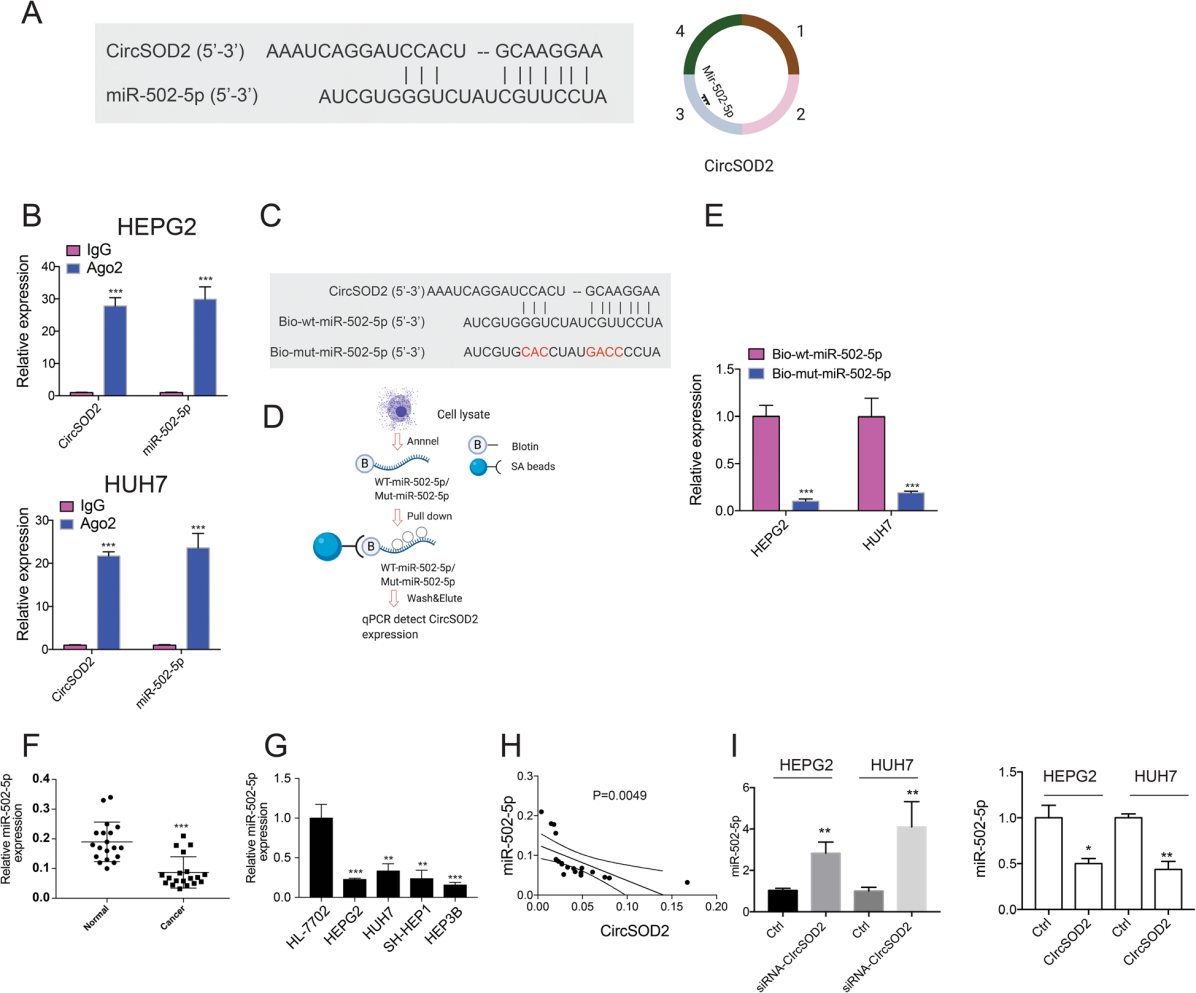
CircSOD2 suppress miR-502-5p expression by acting as a sponge. **a** Diagram of circSOD2 and miR-502-5p interaction sites. **b** Pull down circSOD2 and miR-502-5p with Ago2, IgG was used as control. **c** Diagram of biotin labeled wild type miR-502-5p and mutant miR-502-5p. Mutated sites were labeled with red. **d** Diagram of biotin pull down assay. **e** Detection of circSOD2 expression after pulling down with biotin labeled wild type miR-502-5p or mutant miR-502-5p. **f** miR-502-5p expression in 19 HCC patient tumor tissues and adjacent normal liver tissues. **g** miR-502-5p expression in normal liver cell HL-7702 and liver cancer cell line HEPG2, HUH7, SK-HEP1, and HEP3B. **h** Correlation analysis of miR-502-5p and circSOD2 in HCC patient tumor tissues. **i** miR-502-5p expression after silencing or over-expressing circSOD2. *P < 0.05, **P < 0.01, ***P < 0.001

More and more evidences suggest that, by acting as a sponge, circular RNAs regulate miRNA expression [29, 30]. By searching the circular RNA interactome database, we found that miR-502-5p is a target of circSOD2. The potential interaction site between circSOD2 exon 3 and 5′ miR-502-5p was shown (Fig. 4a). Our previous results showed that Ago2 interacts with circSOD2 in cells. In addition to that, here, we found Ago2 co-precipitated circSOD2 and miR-502-5p from HEPG2 and HUH7 cells (Fig. 4b). To further determine if circSOD2 could directly interact with miR-502-5p. Wild type miR-502-5p or circSOD2-miR-502-5p interaction sites mutated miR-502-5p was labeled with biotin and used to pull down circSOD2 from cell lysates (Fig. 4c-d). As indicated, wild type miR-502-5p efficiently precipitate circSOD2. However, the interaction between mutant miR-502-5p and circSOD2 was almost completely abolished (Fig. 4e). Thus, these results confirmed the direct interaction between circSOD2 and miR-502-5p within liver cancer cells.

To further understand if circSOD2 plays a role in regulating miR-502-5p expression. The expression of miR-502-5p in HCC tumor tissues and liver cancer cells was first investigated. In contrast to circSOD2, miR-502-5p expression was downregulated in HCC patient tumor tissues and liver cancer cell lines compared with normal liver tissues and normal liver cells (Fig. 4f-g). In addition, the expression level of miR-502-5p was negatively correlated with circSOD2 in HCC tumor tissues (Fig. 4h). Furthermore, siRNA mediated circSOD2 knockdown greatly upregulated miR-502-5p expression in the meantime, over-expression of circSOD2 suppressed miR-502-5p expression (Fig. 4i). Above all, these results clearly demonstrate that, by acting as a sponge, circSOD2 suppressed miR-502-5p expression in liver cancer cells.

### Overexpression of miR-502-5p downregulates DNMT3a expression

**Fig. 5:**
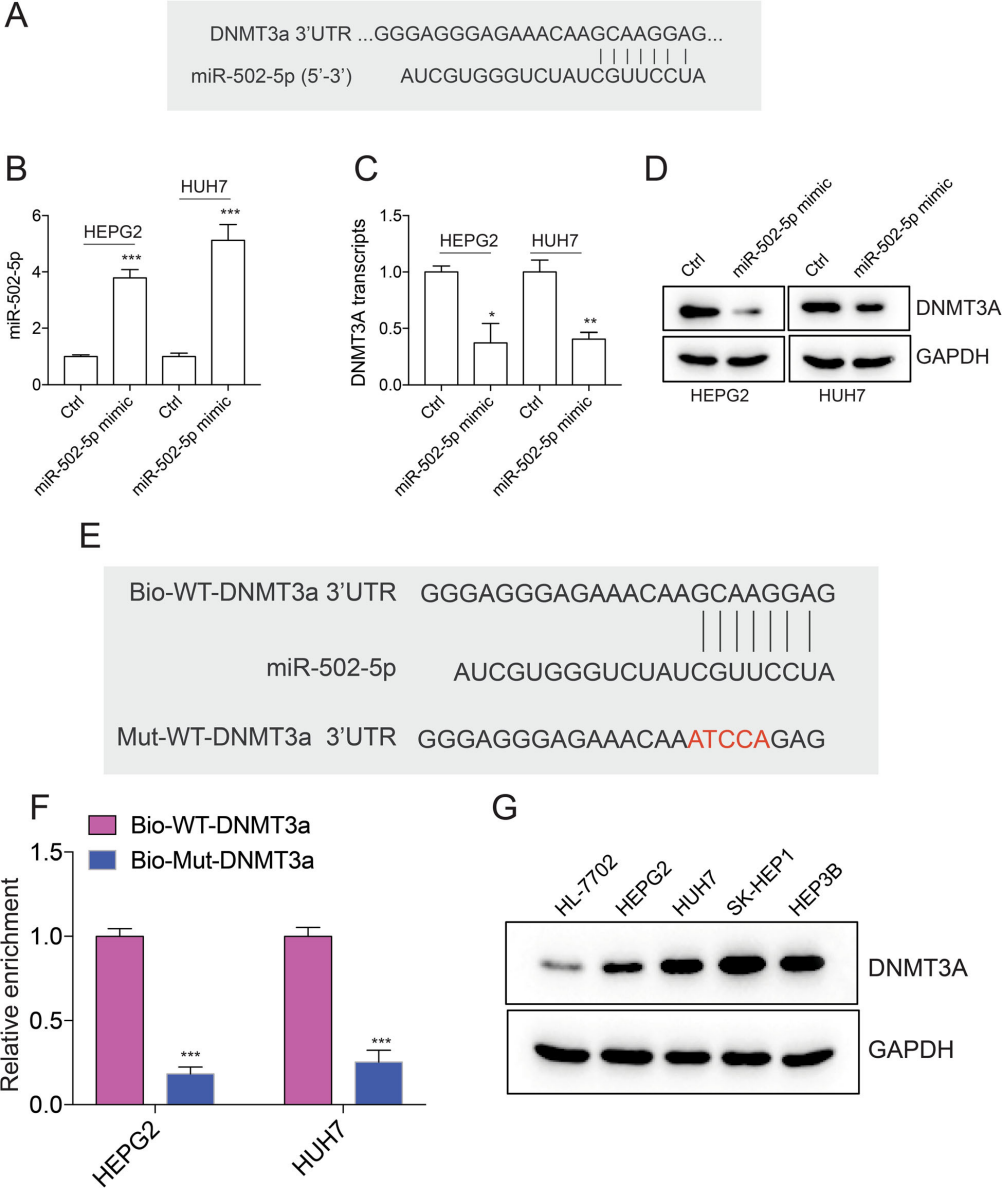
Overexpression of miR-502-5p downregulates DNMT3a expression. **a** Diagram of miR-502-5p and DNMT3a 3’UTR interaction sites. **b** miR-502-5p expression after introducing miR-502-5p mimic into HEPG2 and HUH7 cells. Detection of DNMT3a mRNA (**c**) and protein (**d**) levels after introducing miR-502-5p mimic into HEPG2 and HUH7 cells. **e** Diagram of biotin labeled wild type DNMT3a 3’UTR and mutant DNMT3a 3’UTR. Mutated sites were labeled with red. **f** Detection of miR-502-5p expression after pulling down with biotin labeled wild type DNMT3a 3’UTR or mutant DNMT3a 3’UTR. **g** DNMT3a expression in normal liver cell HL-7702 and liver cancer cell line HEPG2, HUH7, SK-HEP1, and HEP3B. *P < 0.05, **P < 0.01, ***P < 0.001

miRNAs interact with the 3′ untranslated region (3′ UTR) of target mRNAs to induce mRNA degradation and translational repression [31]. Database from miRSystem, and TargetScan suggest that DNMT3a, a DNA methyltransferase is a target of miR-502-5p. The interaction site between DNMT3a 3’UTR and miR-502-5p was shown (Fig. 5a). To test if miR-502-5p regulates DNMT3a expression in liver cancer cells, miR-502-5p mimic was introduced into HEPG2 and HUH7 cells (Fig. 5b). Indeed, introducing miR-502-5p mimic into HEPG2 and HUH7 cells significantly downregulated DNMT3a transcription (Fig. 5c), DNMT3a translation was also repressed (Fig. 5d). To confirm the direct interaction between DNMT3a 3’UTR and miR-502-5p. Wild type or mutant DNMT3a 3’UTR was labeled with biotin and used to pull down miR-502-5p from cell lysates. Wild type DNMT3a 3’UTR significantly enriched miR-502-5p compared to mutant DNMT3a 3’UTR (Fig. 5e-f). The higher DNMT3a expression in liver cancer cells compared to normal liver cells (Fig. 5g) suggests that downregulation of miR-502-5p in HCC may otherwise facilitate DNMT3a expression.

### DNMT3a activates JAK2/STAT3 signaling pathway by suppressing SOCS3 expression

**Fig. 6:**
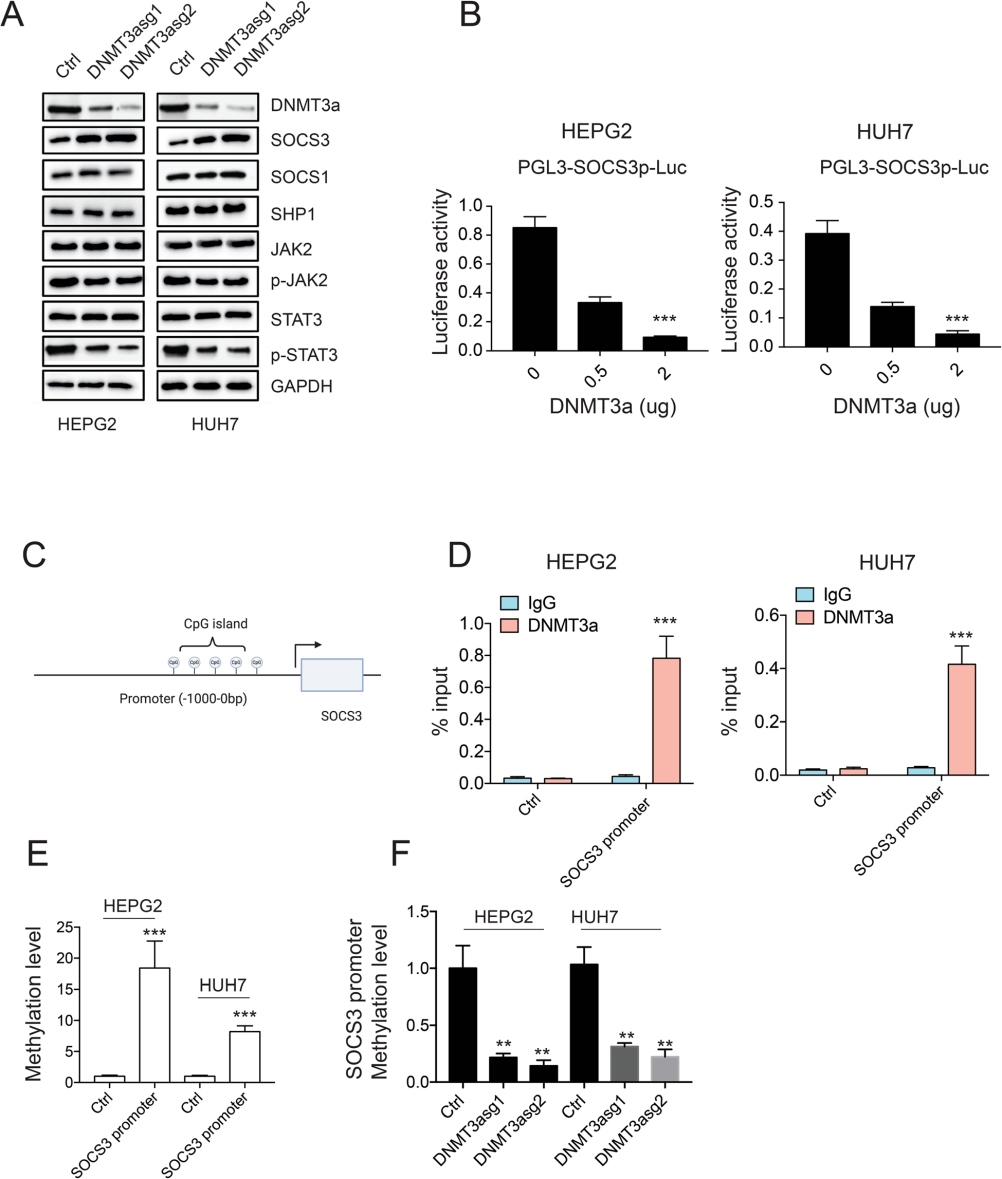
DNMT3a activates JAK2/STAT3 signaling pathway by suppressing SOCS3 expression. **a** Western blot results of genes involved in JAK/STAT3 signaling pathway after deletion of DNMT3a. **b** PGL3-SOCS3 promoter was co-transfected with different doses of MYC-DNMT3a, the activity of SOCS3 promoter was measured by luciferase. Renilla was used as control. **c** Diagram of CpG island in SOCS3 promoter. **d** ChIP-qPCR results of DNMT3a on SOCS3 promoter and its nearby non-CpG region. IgG was used as control. **e** Detection of SOCS3 promoter methylation with Methylated-DNA IP Kit. **f** Detection of SOCS3 promoter methylation after depleting DNMT3a. *P < 0.05, **P < 0.01, ***P < 0.001

Dysregulation of IL-6/STAT3 signaling pathway has been implicated in the pathogenesis of HCC [32–34]. To gain insights into the molecular mechanism underlying the role of DNMT3a in HCC, CRISPR-CAS9 was used to delete DNMT3a from HEPG2 and HUH7 cells, the effect of DNMT3a depletion on the expression of genes involved in JAK/STAT3 signaling pathway was examined. DNMT3a was efficiently deleted by CAS9 in HEPG2 and HUH7 cells. Interestingly, JAK2 inhibitor SOCS3 was greatly upregulated, while phosphorylated JAK2, and phosphorylated STAT3 were all downregulated (Fig. 6a), indicating DNMT3a may be involved in SOCS3/pJAK2/pSTAT3 signaling pathway regulation in HCC. Since SOCS3 locates in the upstream of JAK/STAT signaling pathway. We next asked if DNMT3a could directly regulate SOCS3. Indeed, co-transfecting DNMT3a and SOCS3 promoter driven luciferase vector suppressed luciferase activity in a dose dependent manner (Fig. 6b). However, no effect was observed in SHP1 or SOCS1 promoter activity in the presence of DNMT3a (Supplemental figure 1C-D). DNMT3a is a DNA methyltransferase that modifies CpG methylation and suppresses gene expression. By analyzing SOCS3 promoter, we found that there is an CpG island in the SOCS3 promoter (Fig. 6c), suggesting that DNMT3a may downregulate SOCS3 expression by modifying its promoter methylation status. To test this hypothesis, DNMT3a ChIP-qPCR and DNA methylation pull down assay were performed. DNMT3a was highly enriched in SOCS3 promoter compared to its nearby non-CpG region (Fig. 6d), 5mc antibody also significantly precipitated SOCS3 promoter compared to control (Fig. 6e). Moreover, knocking down DNMT3a decreased SOCS3 promoter methylation in both HEPG2 and HUH7 cells (Fig. 6f). In summary, these results suggest that, in liver cancer cells, DNMT3a upregulation promotes SOCS3 promoter methylation and suppresses SOCS3 expression, which further activates JAK2/STAT3 signaling pathway.

### DNMT3a rescues liver cancer cell proliferation impaired by circSOD2 depletion

**Fig. 7:**
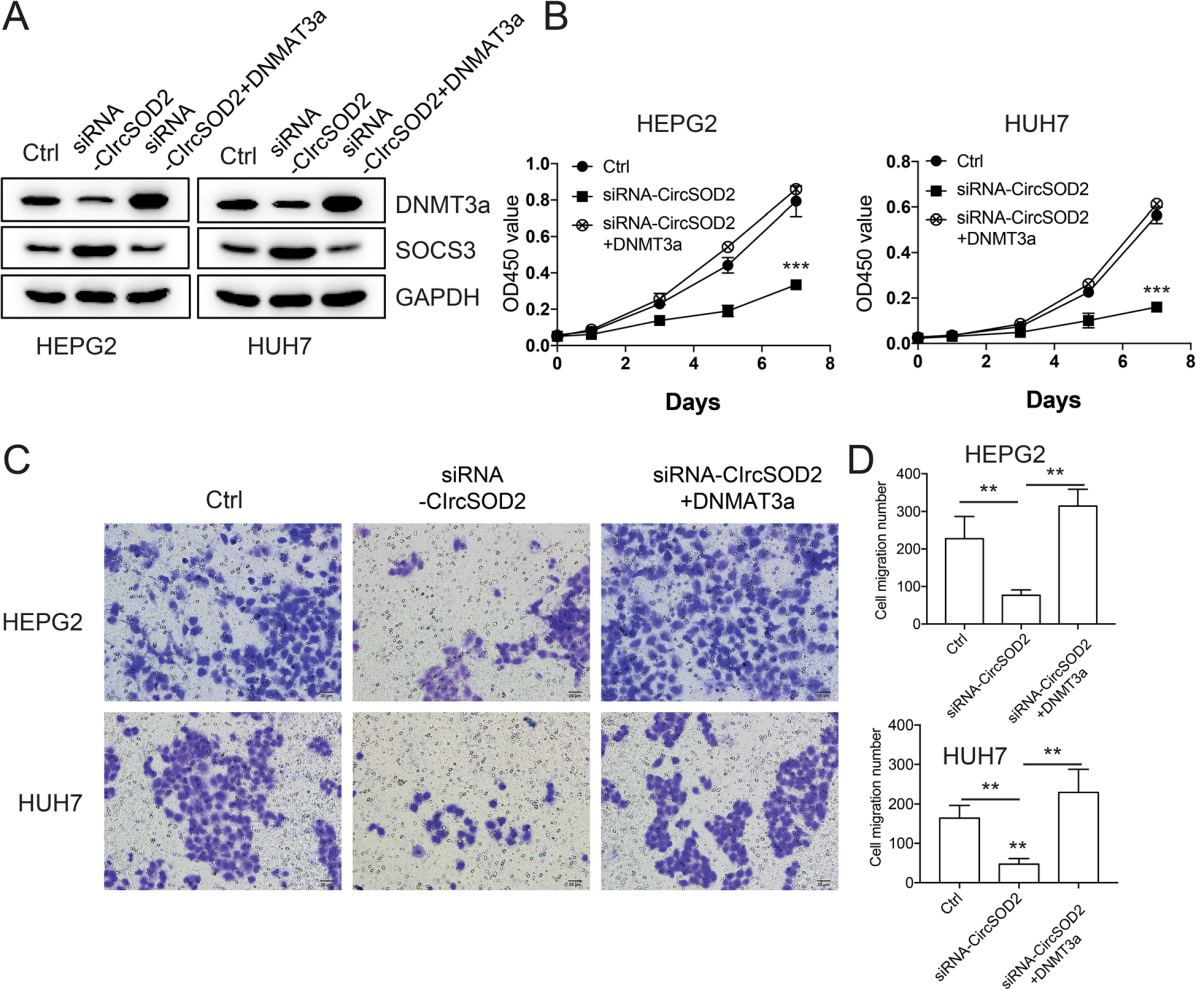
DNMT3a rescues liver cancer cell proliferation impaired by circSOD2 depletion. **a** Western blot results of DNMT3a, SOCS3 after silencing circSOD2 in the presence or absence of DNMT3a, GAPDH was used as control. Cell growth (**b**) and cell migration (**c**) after silencing circSOD2 in the presence or absence of DNMT3a. **d** Statistic results of cell migration. *P < 0.05, **P < 0.01, ***P < 0.001

To further confirm that DNMT3a is the downstream gene among circSOD2 regulated signaling pathway. CircSOD2 was depleted in the presence or absence of exogenously expressed DNMT3a. In the absence of DNMT3a, depletion of circSOD2 downregulated DNMT3a and upregulated SOCS3 expression. However, SOCS3 expression was restored when DNMT3a was expressed (Fig. 7a), suggesting that the effect of circSOD2 depletion on upregulating SOCS3 expression was mediated through suppressing DNMT3a. In line with these results, we showed that the expression of circSOD2 was negatively associated with SOCS3 while positively associated with DNMT3a expression in HCC tissues (Supplemental figure 1). In addition to that, we found that hampered liver cancer cell growth and cell migration induced by circSOD2 depletion were also rescued by DNMT3a (Fig. 7b-d). Collectively, these results demonstrate that DNMT3a is the downstream gene of circSOD2 regulated signaling pathway.

### STAT3 upregulates circSOD2 expression in a feedback way

**Fig. 8:**
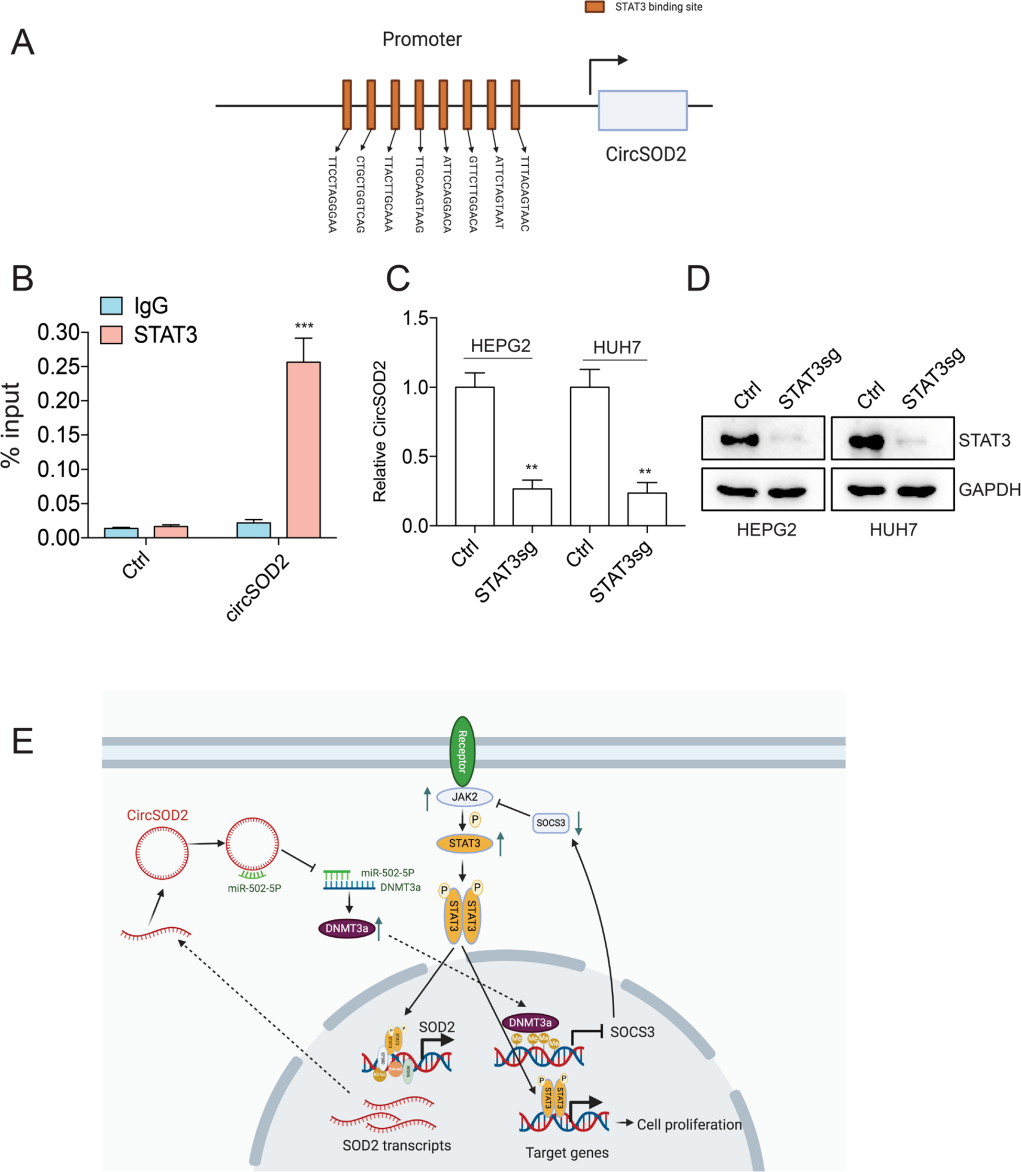
STAT3 upregulates circSOD2 expression in a feedback way. **a** Diagram of STAT3 binding sites on circSOD2 promoter. **b** ChIP-qPCR results of STAT3 binding to circSOD2 promoter. DNA region that is close to circSOD2 promoter without STAT3 binding site and IgG were used as control. **c** CircSOD2 expression after deleting STAT3. **d** Western blots detect STAT3 expression after deleting STAT3, GAPDH was used as control. **e** A schematic model of the mechanism underlying the role of circSOD2 in HCC. *P < 0.05, **P < 0.01, ***P < 0.001

Motif analysis with JASPAR found that eight STAT3 binding sites occupy circSOD2 promoter (Fig. 8a), suggesting that STAT3 may regulate circSOD2 expression. To confirm this, ChIP-qPCR with STAT3 antibody was performed. DNA region that is close to circSOD2 promoter without STAT3 binding sites was used as a control. STAT3 antibody significantly enriched circSOD2 promoter compared to control IgG and control region (Fig. 8b). Depletion of STAT3 also suppressed circSOD2 expression (Fig. 8c-d). These results suggest that STAT3 regulates circSOD2 expression in a feedback way.

### Discussion

Liver cancer poses great threat to human health. Annually, approximately 800,000 new cases are diagnosed with and over 700,000 deaths are linked to liver cancer worldwide [1]. Understanding its underlying pathogenesis mechanism may provide therapeutic targets. In this study, we found that circSOD2, derived from SOD2 was highly expressed in HCC patient tumor tissues and liver cancer cells compared to normal liver tissues and normal liver cells, respectively. Depletion of circSOD2 significantly ceased liver cancer cell growth and impaired in vivo tumorigenesis. Mechanically, we showed that, by acting as a sponge for miR-502-5p, circSOD2 suppressed miR-502-5p expression, which in turn upregulated DNMT3a expression. Elevated DNMT3a suppressed SOCS3 expression and further activates JAK2/STAT3 signaling pathway. In addition, we also found that increased STAT3 in liver cancer cells also regulates circSOD2 expression in a feedback way.

Complexed roles of miR-502 have been revealed in different cancers. For example, hsa-miR-502-5p (miR-502) was reported to inhibit autophagy and tumor growth in colon cancer by suppression of Rab1B [35]. Exogenously introduce miR-502-5p mimic into breast cancer cells enhanced early cell apoptosis and inhibited cell proliferation [36]. However, upregulation of miR-502 has been reported to accelerate esophageal cancer cell TE1 proliferation by promoting AKT phosphorylation [37]. In this study, we found that, similar to breast cancer and colon cancer, miR-502-5p was also downregulated in HCC. Further studies on circSOD2 and miR-502-5p showed that miR-502-5p binds to circSOD2 exon 3, depletion of circSOD2 significantly upregulated miR-502-5p expression. These results suggest that in HCC, miR-502-5p was suppressed by circSOD2 through direct interaction.

Bioinformatic analysis showed that miR-502-5p binds to DNMT3a 3’UTR. Introducing miR-502-5p into liver cancer cells greatly suppressed DNMT3a expression, suggesting that DNMT3a is a target of miR-502-5p in HCC. The importance of DNMT3a in HCC progression has been extensively studied. Zhao et al., found that depletion of DNMT3a suppressed HCC cell proliferation [38]. Chen et al., reported that miR-30a-3p inhibits the proliferation of liver cancer cells by targeting DNMT3a [39]. Similarly, in this study we also demonstrated that downregulation of DNMT3a by silencing circSOD2 impaired liver cancer cell growth and migration.

DNMT3a mediated DNA hypermethylation suppressed gene expression and has been associated with many cancer developments. DNA hyper-methylation at NF2 and KIBRA promoter mediated by MOC2 and DNMT3a complexes suppresses their gene expression and Hippo signaling pathway activation, which further promotes HCC cancer stemness and tumorigenesis [40]. In Triple Negative Breast Cancer, coupled with MYC, DNMT3a promotes the epithelial to mesenchymal transition and mammosphere formation of TNBC cells by upregulating miR-200b promoter methylation [41]. SUV39H1/DNMT3A-dependent methylation of the RB1 promoter stimulates PIN1 expression and melanoma development [42]. Here, by knocking down DNMT3a, we discovered that SOCS3 is regulated by DNMT3a. Further analysis and experimental validation demonstrated that upregulated DNMT3a in liver cancer cells promoted SOCS3 promoter hyper-methylation and suppressed SOCS3 expression.

Abnormal activation of IL-6/STAT3 signaling pathway has been implicated in the progression of HCC. In HBx induced hepatocellular carcinoma mice model, IL-6/STAT3, and Wnt/β-catenin signaling pathways are constitutively activated [43]. Tumor cell-intrinsic Tim-3 promotes liver cancer via NF-κB/IL-6/STAT3 axis [34]. Long non-coding RNA DILC regulates liver cancer stem cells via IL-6/STAT3 axis [32]. In this study, we uncovered a new mechanism through which STAT3 is activated, we showed that, SOCS3 suppression mediated by DNMT3a methylation, activated JAK2/STAT3 signaling pathway. Due to the complexity of IL-6/STAT3 signaling pathway, more studies should be performed to clarify the crosstalk among different pathways.

We also evaluated the mechanism through which circSOD2 was activated in HCC. We found that by tethering to circSOD2 promoter, EP300 and WDR5 facilitated circSOD2 promoter H3K27ac and H3K4me3 modification respectively, and increased circSOD2 expression. Moreover, STAT3 binding sites on circSOD2 promoter recruited STAT3 binding to circSOD2 promoter and upregulated circSOD2 expression in a feedback way. Although, for now, we do not have any idea whether STAT3 recruits EP300 or WDR5 into circSOD2 promoter or vice versa, more studies should be performed in the future to clarify this.

## Conclusions

In summary, our clinical data, in vitro cell assay, and tumor formation support the notion that circSOD2 plays a critical role in HCC progression. circSOD2/miR-502-5p/DNMT3a/SOCS3-JAK2/STAT3/circSOD2 axis, a novel pathway through which JAK2/STAT3 is activated provides promising therapeutic targets against HCC.

## Supplementary Information

**Additional file 1:Supplemental Table 1.** Clinicopathological characteristics of HCC patients (*n* = 19).

**Additional file 2:Supplemental Figure 1.** H3K27ac and H3K4me3 modification on circSOD2 promoter in liver cancer cell and normal liver cell. (A) ChIP-qPCR results of H3K27ac enrichment on circSOD2 promoter in liver cancer cell HEPG2, HUH7 and normal liver cell HL-7702. (B) ChIP-qPCR results of H3K4me3 enrichment on circSOD2 promoter in liver cancer cell HEPG2, HUH7 and normal liver cell HL-7702. (C) PGL3-SHP1 or PGL3-SOCS1 (D) promoter was co-transfected with different doses of MYC-DNMT3a, the activity of SHP1 or SOCS1 promoter was measured by luciferase. Renilla was used as control. (E) The correlation of circSOD2 and SOCS3 in HCC tissues. (F) The correlation of circSOD2 and DNMT3a in HCC tissues. *P < 0.05, **P < 0.01, ***P < 0.001.

## Data Availability

The datasets used and/or analyzed during the current study were available from the corresponding authors on reasonable request.

## Abbreviations

MiRNA: Micro RNA
LncRNA: Long non-coding RNA
HCC: Hepatocellular carcinoma
ChIP-qPCR: Chromatin Immunoprecipitation quantitative real time PCR
CircRNA: Circular RNA
qRT-PCR: Real-Time Quantitative Reverse Transcription PCR
PI: Propidium iodide
RIP: RNA immunoprecipitation
NC: Nitrocellulose membrane
RIPA: Radioimmunoprecipitation assay
3′ UTR: 3′ untranslated region
