# Supplementary material for: CircSOD2 induced epigenetic alteration drives hepatocellular carcinoma progression through activating JAK2/STAT3 signaling pathway
Source: J Exp Clin Cancer Res. 2020 Nov 25;39:259. doi: 10.1186/s13046-020-01769-7 (PMC7687771; doi:10.1186/s13046-020-01769-7)
Supplement: Supplementary file 1 — Additional file 1:Supplemental Table 1. Clinicopathological characteristics of HCC patients (n = 19). [file 13046_2020_1769_MOESM1_ESM.docx]

**Supplemental table 1.** Clinicopathological characteristics of HCC patients (n=19)

| Characteristic | Cases | cirSOD2 expression | | P-value |
| --- | --- | --- | --- | --- |
|  |  | Low N (%) | High N (%) |  |
| Age (years) |  | 12 | 7 |  |
| <50 | 11 | 7 (58.3%) | 4 (57.1%) | 0.9596 |
| ≥50 | 8 | 5 (41.7%) | 3 (42.9%) |  |
| Gender |  |  |  |  |
| male | 12 | 8 (66.7%) | 4 (57.1%)) | 0.678 |
| female | 7 | 4 (66.7%) | 3 (42.9%)) |  |
| Histological grade |  |  |  |  |
| I/II | 9 | 8 (66.7%) | 1 (14.3%) | *0.0274 |
| III | 10 | 4 (66.7%) | 6 (85.7%) |  |
| Tumor size |  |  |  |  |
| <5 cm | 8 | 5 (41.7%)) | 3 (42.9%)) | 0.9596 |
| ≥5 cm | 11 | 7 (58.3%) | 4 (57.1%)) |  |
