# Supplementary figures and images for: CircSOD2 induced epigenetic alteration drives hepatocellular carcinoma progression through activating JAK2/STAT3 signaling pathway
Source: J Exp Clin Cancer Res. 2020 Nov 25;39:259. doi: 10.1186/s13046-020-01769-7 (PMC7687771; doi:10.1186/s13046-020-01769-7)

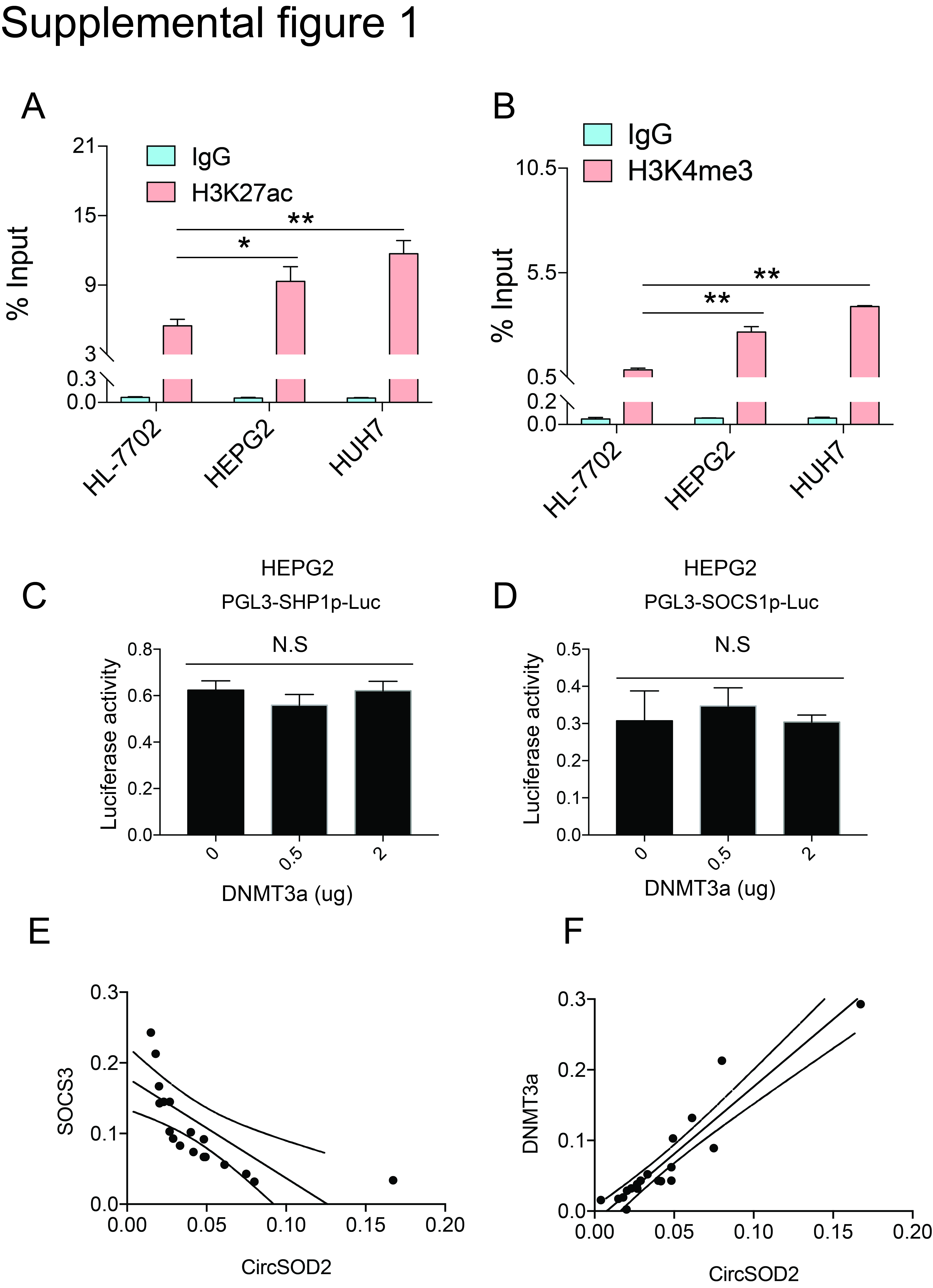

Supplement: Supplementary file 2 — Additional file 2:Supplemental Figure 1. H3K27ac and H3K4me3 modification on circSOD2 promoter in liver cancer cell and normal liver cell. (A) ChIP-qPCR results of H3K27ac enrichment on circSOD2 promoter in liver cancer cell HEPG2, HUH7 and normal liver cell HL-7702. (B) ChIP-qPCR results of H3K4me3 enrichment on circSOD2 promoter in liver cancer cell HEPG2, HUH7 and normal liver cell HL-7702. (C) PGL3-SHP1 or PGL3-SOCS1 (D) promoter was co-transfected with different doses of MYC-DNMT3a, the activity of SHP1 or SOCS1 promoter was measured by luciferase. Renilla was used as control. (E) The correlation of circSOD2 and SOCS3 in HCC tissues. (F) The correlation of circSOD2 and DNMT3a in HCC tissues. *P < 0.05, **P < 0.01, ***P < 0.001. [file 13046_2020_1769_MOESM2_ESM.tif]
